# Assessment of Exercise-Induced Dehydration Status Based on Oral Mucosal Moisture in a Field Survey

**DOI:** 10.3390/dj13010005

**Published:** 2024-12-25

**Authors:** Gen Tanabe, Tetsuya Hasunuma, Yasuo Takeuchi, Hiroshi Churei, Kairi Hayashi, Kaito Togawa, Naoki Moriya, Toshiaki Ueno

**Affiliations:** 1 Department of Sports Dentistry, School of Dentistry, Meikai University, Sakado 350-0283, Japan; gen.spmd@dent.meikai.ac.jp (G.T.); kaito321@dent.meikai.ac.jp (K.T.); 2 Department of Masticatory Function and Health Science, Graduate School of Medical and Dental Sciences, Institute of Science Tokyo, Tokyo 113-8510, Japan; chu.spmd@tmd.ac.jp (H.C.); k.hayashi.spmd@tmd.ac.jp (K.H.); 3 Faculty of Human Development and Culture, Fukushima University, Fukushima 960-1296, Japan; hasunuma@educ.fukushima-u.ac.jp (T.H.); moriya@bunka.ac.jp (N.M.); 4 Public Interest Incorporated Association, Japan Triathlon Union, Tokyo 160-0013, Japan; 5 Department of Lifetime Oral Health Care Science, Graduate School of Medical and Dental Sciences, Institute of Science Tokyo, Tokyo 113-8510, Japan; takeuchi.peri@tmd.ac.jp; 6 Bunka Gakuen University, Tokyo 151-8523, Japan

**Keywords:** dehydration, sweat rate, oral mucosal moisture, saliva flow rate, secretory immunoglobulin A (s-IgA), hydration assessment, capacitance sensor

## Abstract

**Background/Objective:** Conventional techniques for evaluating hydration status include the analysis of blood, urine, and body weight. Recently, advancements in dentistry have introduced capacitance sensor-based oral epithelial moisture meters as promising avenues for assessment. This study aimed to examine the correlation between oral mucosal moisture content, as determined using a capacitance sensor, and exercise-induced dehydration. **Methods:** A total of 21 participants engaged in a 120 min slow distance exercise session. A series of measurements were taken before and after the exercise session, including body weight, sweat rate, secretory immunoglobulin A (s-IgA) concentration in saliva samples, saliva flow rate, and oral mucosal moisture content, which were assessed using a capacitance sensor. The relationship between physical dehydration and oral mucosal moisture content was investigated using statistical analysis. Receiver operating characteristic curves were constructed to ascertain whether variations in oral mucosal moisture content could discern body mass losses (BMLs) of 1.5% and 2%. **Results:** A significant correlation was observed between the sweat rate during exercise and the change in oral mucosal moisture content before and after exercise (Spearman’s rank correlation coefficient: ρ = −0.58, *p* < 0.001). The salivary flow and s-IgA secretion rates were lower after the exercise period than before, whereas the s-IgA concentration was higher. Oral mucosal moisture decreased during the exercise period. Receiver operating characteristic curve analysis revealed that differences in oral mucosal moisture content exhibited discriminative capabilities, with area under the curve values of 0.79 at 1.5% BML and 0.72 at 2% BML. **Conclusions:** The measurement of oral mucosal moisture using capacitance sensors represents a promising noninvasive approach for the assessment of exercise-induced dehydration.

## 1. Introduction

Water is the principal component of the human body and is essential for optimal physiological health and function. To maintain water balance in the body, humans must drink an adequate amount of water each day. Heat exposure during exercise can significantly increase thermal sweat loss. Sweat evaporation primarily disperses heat from the body, resulting in the loss of both water and electrolytes. Water and electrolyte imbalances (dehydration and hyponatremia) can adversely affect anaerobic workout [1], energy metabolism [2], cognition [3], and overall health. Aerobic exercise [4] and cognitive/mental [3] performance are impaired when dehydration exceeds 2% of body weight. Furthermore, >3% dehydration reduces muscle strength [5]. Additional effects of dehydration include severe heat exhaustion [6], exertional heat stroke [7], rhabdomyolysis [8], and exercise-associated hyponatremia [9]. In severe cases, dehydration can be fatal; therefore, prompt evaluation is critical.

Blood, urine, and body weight have been studied as determinants of dehydration [10]. In sports, urine indices are used because of their practicality; in particular, urine-specific gravity is a noninvasive, inexpensive, simple, fast, and accurate indicator of hydration status before exercise [11]. Oral characteristics such as wrinkles, dryness of the mouth, and subjective sensations of thirst in the oral cavity and/or throat have also been used to assess dehydration. Additional diagnostic indicators include salivary flow, which decreases with dehydration after exercise [12], salivary viscosity [13], salivary osmotic pressure [14], and capillary refill time [15].

The thirst in the mouth and/or throat is a subjective afferent sensation that triggers alcohol consumption. It has been studied not only as an element of dehydration status but also as a means of promoting recovery from water loss. McGee et al. [16] have reported that 85% of patients with dehydration experienced dry mouth. The thirst that prompts spontaneous drinking behaviors is categorized as physiological or perceived. Physiological thirst is a hypomoist uremic signal perceived at the cellular level and transmitted via the central nervous system. Perceived thirst, which is recognized by oropharyngeal factors in the mucosa, has a higher threshold than physiological thirst and is delayed by dehydration [10,17]. Subjective thirst in the mouth and/or throat is directly related to dehydration of the entire body; however, it has low sensitivity and provides limited information for assessing dehydration.

In recent years, research and development in dental science have focused on the application of capacitance sensor-based oral epithelial moisture meters to quantify xerostomia and thirst in the mouth. Emerging uses of oral epithelial moisture measurements include the evaluation of xerostomia [18] and oral dryness in older populations [19], as well as in patients with maxillofacial prostheses [20,21] or oropharyngeal cancer [22]. Expanding insight into the physiological implications of oral mucosal moisture beyond xerostomia, clinicians have begun using capacitance sensor-based oral epithelial moisture meters to evaluate dehydration. A previous study has documented the usefulness of capacitance sensors for the initial assessment of the dehydration status of patients transported to emergency departments for acute treatment [23].

In the field of sports, there are various indices of dehydration, largely divided into acute and chronic indices [1]; however, the acute real-time dehydration index is sought as an indicator of dehydration during sports activities. Acute indicators and available samples include total body water and blood; however, these are not freely measurable. In this context, the mouth and thirst indices that have been used as adjuncts to acute indices, although simple, are subjective assessments of the athletes themselves and lack reliability. To overcome this problem, an immediate evaluation method using an instrument to measure the degree of oral mucosal moisture for the quantitative diagnosis of xerostomia was developed. The capacitance oral epithelial moisture sensor used in this study has no restrictions on who can use it, and anyone can easily use it. It is small and easy to perform. The measurement time was brief (only a few seconds), and the burden on the athlete and measurer was minimal, suggesting that the device would be well suited for use in sports. However, the verification of the dehydration detection capability of this device remains necessary.

Previous validation studies using capacitance sensors to assess dehydration during exercise and to correlate dehydration with urine-specific gravity [24] have indicated that oral mucosal moisture could be a straightforward indicator for detecting dehydration. This was evidenced by the subjects’ urine specific gravity falling within the range of 1.001–1.032 g/mL, with higher values indicating dehydration, encompassing both normal and dehydrated states. A moderate negative correlation was observed between oral mucosal moisture and urine-specific gravity (*p* < 0.05, r = −0.69). As this field-based report was based on early morning fixed-point observations in an athlete training camp, the reliability of the capacitance sensors before and after exercise, as well as their relationship with other dehydration indicators, remains unknown. Therefore, the purpose of this study was to test the hypothesis that oral moisture measurements obtained using capacitance sensors could rapidly and accurately correlate and detect dehydration before and after physical exercise. The relationship between physical dehydration and oral mucosal moisture content was evaluated using capacitance sensors. A clinical exercise intervention study was conducted during marathon training sessions.

## 2. Materials and Methods

### 2.1. Study Participants

Using a previously calculated effect size [24], a sample size of 20 was required for the present study to achieve a statistical power of 0.80 with an alpha level of 0.05. The inclusion criteria were individual training for a full marathon and no known cardiovascular disease, diabetes mellitus, Sjögren syndrome, xerostomia, or oral mucosal abnormalities of the tongue. Upon arrival at the training site, the participants attended a research briefing, after which they signed a written informed consent form and were instructed to avoid consuming alcohol and caffeine the day (24 h) before the test, considering the half-life of alcohol and caffeine metabolism in the body.

The study protocol was approved by the Research Ethics Committee of Tokyo Medical and Dental University (approval no: D2019-031/2 Feb 2020), and the experiments were conducted in accordance with the Declaration of Helsinki.

### 2.2. Experimental Design and Data Collection

Each participant completed 120 min of long slow distance (LSD) training outdoors, effectively simulating real-world endurance activities during winter. The participants were free to wear long sleeves or pants for training. Weather and wet-bulb globe temperatures (WBGTs) were recorded to document the exercise environment. The maximum heart rate, distance run (determined using a wristwatch; Garmin, Ltd., Schaffhausen, Switzerland), and rate of perceived exertion (based on the Borg scale) were recorded immediately after LSD training. During LSD training, the participants were allowed to drink freely but not eat, and the intensity of the exercise was at the discretion of the individual; therefore, they were instructed so their maximum heart rate did not exceed 180 beats per minute. The participants’ pre- and post-exercise body weights were recorded, as were fluid intake and urine volume during training. The volume of fluid intake was determined by weighing the designated beverages (500 mL) before and after exercise. Body mass loss (BML) was determined by comparing pre- and post-training body weight. The sweat rate was calculated using the following formula:

Sweat rate = [pre-exercise body weight (kg) − post-exercise body weight (kg) + fluid intake (L) − urine volume (L)]/exercise time (h)

Sweat rate data were not adjusted for weight loss associated with energy metabolism or respiratory fluid losses [25,26].

Saliva samples were obtained from the subjects before and after the training sessions. They were directed to perform an intraoral rinse with water and were instructed to refrain from consuming food or beverages. The participants provided an unstimulated saliva sample by passively dropping their saliva into a plastic container for 2 min with minimal orofacial movement and their heads tilted slightly forward [27]. The saliva samples were weighed to measure saliva volume, and assuming a saliva density of 1 g/mL, the saliva flow rate (mL/min) was calculated by dividing the saliva volume by the collection duration. The saliva samples were immediately stored at 4 °C and subsequently at −20 °C for one week until analysis. The samples were thawed and centrifuged at 17,000 × *g* for 2 min, and the supernatants were diluted five-fold in phosphate-buffered saline to measure the salivary biomarker secretory immunoglobulin A (s-IgA) using a salivary secretory IgA ELISA Kit (Salimetrics LLC., Carlsbad, CA, USA). The s-IgA samples were twice analyzed against a standard curve from 0 to 600 µg/mL, with a sensitivity of 2.5 µg/mL. The results were expressed as absolute s-IgA concentration (µg/mL), and the s-IgA secretion rate (µg/min) was calculated by multiplying the s-IgA concentration by the saliva flow rate.

**Figure 1:**
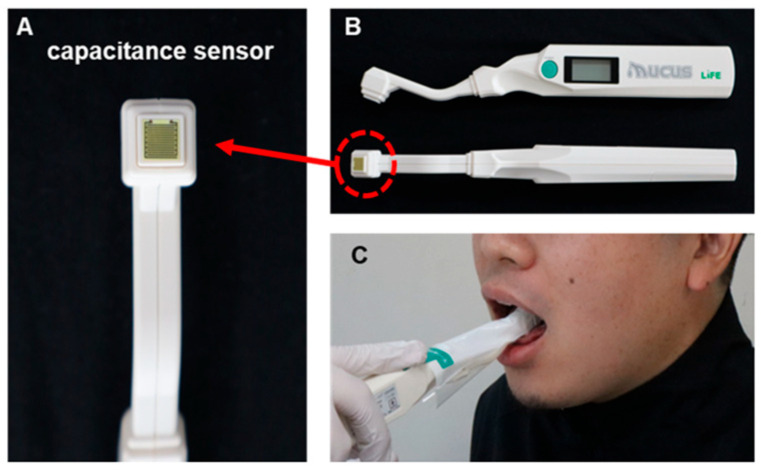
Measuring Mucus^®^ capacitance sensor. (**A**) Sensor section, (**B**) Overall view of sensor, (**C**) scene of measurement.

The amount of oral mucosal moisture was measured using a Mucus^®^ capacitance sensor (Life Co., Ltd., Saitama, Japan; Figure 1), which easily measures oral moisture within 2 s [28]. The moisture content of the substance was determined by calculating the capacitance from the dielectric constant of the substance, using the same principle as that for the skin moisture sensor. Water has a much higher dielectric constant than other substances. Therefore, the percentage of water in a substance can be determined by measuring its dielectric constant. The value (%) obtained using an oral moisture meter was based on the gravimetric moisture content of a standard sample of dry protein membrane designed for medical use and is expressed as a percentage of the moisture content. Moisture content was determined using the following gravimetric method:

Moisture content = B/(A + B) × 100%,

where A is the weight of the dry protein membrane, B is the weight of water, and 100% represents the state in which the sample is entirely composed of water.

The measurement range of the moisture meter was approximately 15–65%. The reliability of the data was previously demonstrated through a comparison with the dry weight method [28]. The oral moisture values range from 0 to 99.9, and values of ≥29.6, 28.0–29.5, and ≤27.9 are defined as normal, borderline dry mouth, and dry mouth, respectively [29].

The capacitor sensors received manufacturing and marketing approval for use as body composition analyzers from the Pharmaceutical and Medical Devices Agency of Japan (approval no. 22200BZX00640000). Before and after the LSD training sessions, oral moisture was measured at the center of the lingual mucosa, approximately 10 mm from the tip of the tongue, with the device manually applied at a pressure of approximately 200 g by a single measurer, as previously described [24]. A dedicated disposable polyethylene cover was attached to the sensor of each participant. The measurements were performed three times, and the median values were recorded [24].

### 2.3. Data Analysis

Statistical analysis was performed using the JMP14 software (SAS Institute Inc., Cary, NC, USA) at a 5% significance level. Data are expressed as mean ± SD (if normally distributed) or median. Body weight, saliva flow rate, s-IgA concentration and secretion rate, and oral mucosal moisture content were compared before and after LSD training using Student’s paired *t*-test/Wilcoxon signed-rank sum test after confirming normality using the Shapiro–Wilk test. Receiver operating characteristic (ROC) curve analysis was performed to determine whether differences in oral mucosal moisture content could detect 1.5% and 2% BML. The area under the curve (AUC) represents the probability that a randomly selected individual would be correctly classified by the diagnostic test. The optimal cutoff value for each hydration variable was determined by determining the highest Younden index value.

## 3. Results

**Figure 2:**
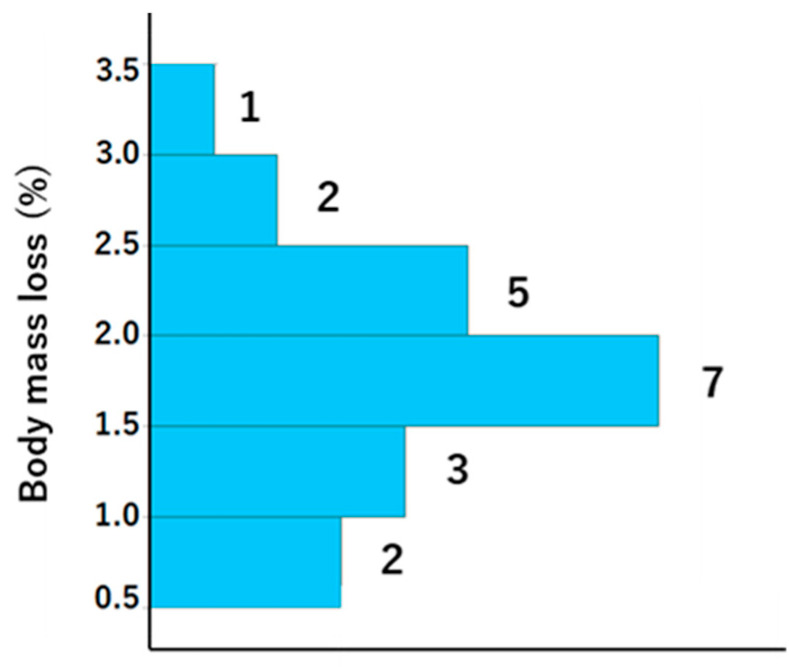
Histogram showing the body mass loss for each participant. The numbers to the right of the bars represent histogram data values.

A total of 21 participants, with ages ranging from 29 to 78 (mean ± standard deviation [SD]: mean 48 ± 12.7), were included in the study, comprising 11 men (age, mean ± SD: 51.3 ± 13.2 years) and 10 women (age, mean ± SD: 44.6 ± 10.8 years). The weather was sunny, and the WBGT was 13.1 °C at the start and 16.2 °C at the end of LSD training; thus, the risk of dehydration and heat stroke was generally assumed to be low. The exercise indices and dehydration assessment data are presented in Table 1 and Table 2, respectively. With regard to exercise load, there were notable individual differences in heart rate and running distance. However, the results of the Borg scale indicate that the exercise load was, to some extent, homogenized among the participants, although it was an internal load. Body weight was lower after vs. before training, and the difference was 1.1 ± 0.5 (Table 2). The BML was 0.5–1.5% in six athletes, 1.5–2% in seven athletes, and >2% in eight athletes (Figure 2).

**Figure 3:**
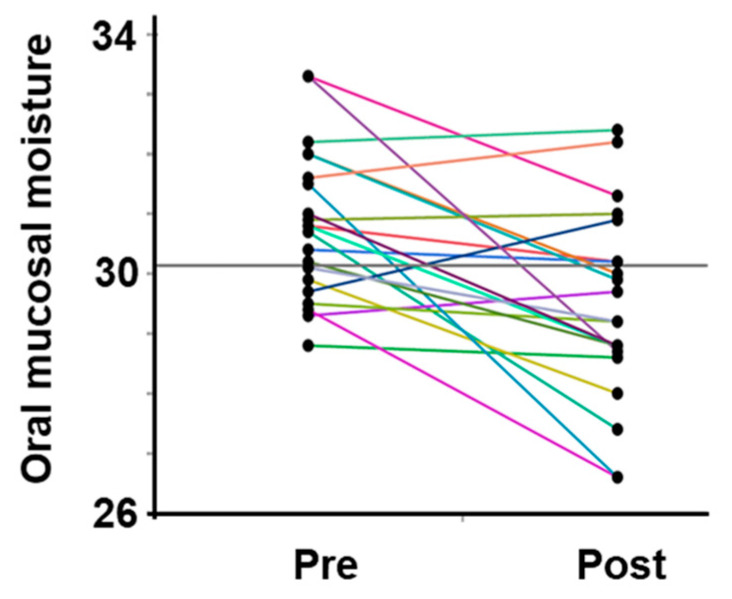
Changes in the oral mucosal moisture content in all participants. The intra-individual data variation is connected by lines. Pre indicates pre-exercise data; post indicates post-exercise data.

**Figure 4:**
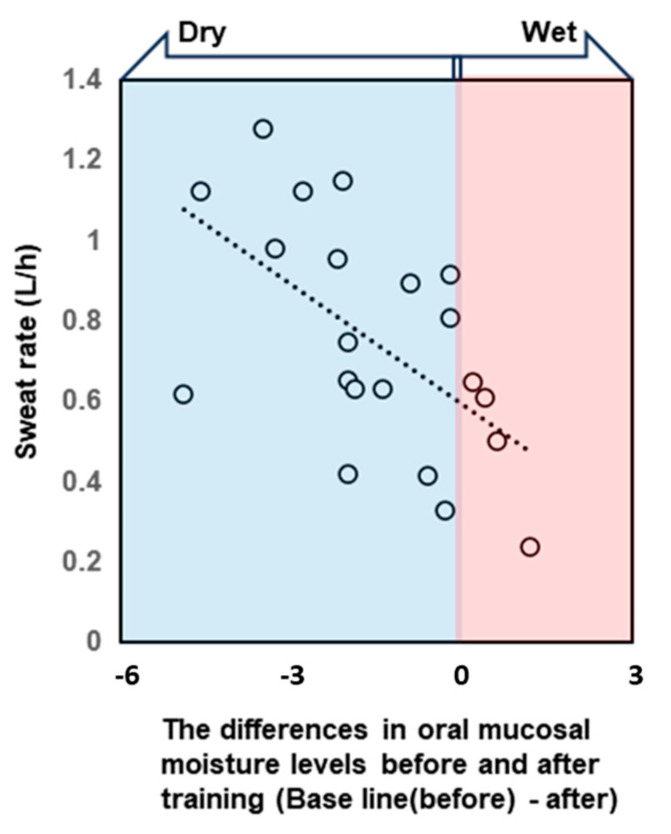
The scatterplot shows the correlation between the sweat rate during training and the difference in oral mucosal moisture content before and after training. Negative difference values indicate that the oral cavity became drier after exercise. Differences in oral mucosal moisture levels before and after training correlated with the post-exercise sweat rate. Spearman’s rank correlation coefficient: ρ = −0.58, *p* < 0.001.

All participants were dehydrated, primarily because of sweating, despite training in a low WBGT environment. Water intake during exercise was 496.9 ± 222.7 mL, and the calculated hydration rates based on the BML were all below 100%. The sweat rate, calculated based on the provided data, was 0.77 ± 0.26 L/h (Table 2). The saliva flow rate was also lower after vs. before exercise, and the oral mucosal moisture content was significantly lower (decrease from 30.8 ± 1.3 to 29.5 ± 1.6) (Figure 3, Table 2). A multivariate correlation analysis of the differences in oral mucosal moisture between pre- and post-training revealed no statistically significant correlation between Perceived exertion (Spearman’s rank correlation coefficient: ρ = −0.07, *p* < 0.74) and the amount of fluid intake (ρ = 0.18, *p* < 0.41); however, a moderate negative correlation was observed running distance (ρ = −0.44, *p* < 0.05). s-IgA, as absolute concentration (µg/mL), was higher after exercise, whereas the s-IgA secretion rate (µg/min) was lower (Table 3). A Shapiro–Wilk test indicated that the data of body weight (before/after; *p* = 0.171/*p* = 0.158), saliva flow rate (*p* = 0.054/*p* = 0.003), s-IgA concentration (*p* = 0.013/*p* = 0.977) and secretion rate (*p* = 0.053/*p* = 0.008), and oral mucosal moisture content (*p* = 0.535/*p* = 0.770) are consistent with a normal distribution, and the subsequent Student’s paired *t*-test/Wilcoxon signed-rank sum test showed a significant difference in body weight, saliva flow rate, and oral mucosal moisture content between before and after LSD training (Table 2). Table 4 shows the results of the multiple regression analysis using the differences in oral mucosal moisture levels before and after training, body weight before training, sex, and pre-training oral mucosal moisture content as factors to confirm the covariates for sweat rate. The results show that only differences in oral mucosal moisture levels before and after training were significant (*p* < 0.05), and there was no interaction between the differences in oral mucosal moisture levels and body weight before training, sex, or pre-training oral mucosal moisture content. The differences in oral mucosal moisture levels before and after training according to sweat rate are shown in Figure 4. The predicted oral mucosal moisture level correlated with the post-exercise sweat rate (Spearman’s rank correlation coefficient: ρ = −0.58, *p* < 0.001).

**Figure 5:**
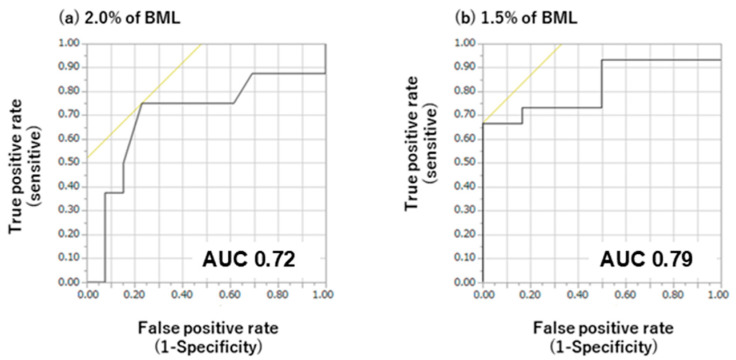
Receiver operating curve (ROC) analysis of the difference in oral mucosal moisture content during exercise at body mass losses (BMLs) of 2% (**a**) and 1.5% (**b**). AUC, area under the ROC curve. Black lines show sensitivity and specificity, and the intersection of the black and yellow lines decides this specific cut-off value. At 1.5% BML, sensitivity and specificity are 0.68 and 0.00 at specific cut-off values, respectively, and an AUC of 0.79. At 2.0% BML, sensitivity and specificity are 0.75 and 0.24% at specific cut-off values, respectively, and an AUC of 0.72.

The criterion values for the differences in oral mucosal moisture content obtained via ROC analysis are presented in Figure 5. Statistical analysis of the ROC curves for varying degrees of BML revealed a significant discriminative capacity for oral mucosal moisture changes. At 1.5% BML, oral mucosal moisture had an AUC of 0.79 with a criterion value of 1.9, thus achieving the highest Younden index value. At 2.0% BML, oral mucosal moisture had an AUC of 0.72 with a criterion value of 2.0, thus achieving the highest Younden index value.

## 4. Discussion

This study tested the hypothesis that oral moisture measurements obtained using capacitance sensors can rapidly and accurately correlate with and detect dehydration before and after physical exercise. In support of this hypothesis, before and after 120 min of LSD training, the participants were dehydrated owing to perspiration, and there was a relationship between sweat rate and oral mucosal moisture content (Figure 4) and the detection of dehydration using capacitance sensors (Figure 5).

Based on a previous measurement, 1–3% BML was expected, but the magnitude of dehydration depended on each athlete’s individual response to the exercise load. The sweat rate, the most important indicator of dehydration in this study, was 0.77 ± 0.26 L/h. In the report by Baker et al., the sweat rate of approximately 500 athletes typically ranged from approximately 0.5 to 2.0 L/h, with variations among the athletes [30]. Although the sweat rate was slightly lower than expected, it was consistent with values reported in a previous study. In the current study, which was a field survey with nonstandardized subjects, the tests were conducted on sunny winter mornings, in which the WBGT was not high and was always at Stage 1 during the 120 min LSD. Although hot and humid summer data are expected to provide more meaningful data, the ability to detect changes in oral mucosal moisture content caused by dehydration in nonhot environments could potentially be applied to all seasons but not in hot environments and should be considered when interpreting the present study data.

In the current study, the saliva flow rate and oral mucosal moisture content were lower after exercise than before exercise, suggesting that oral mucosal moisture content is correlated with sweat rate. The loss of oral mucosal moisture after exercise provides insights into the nature of dehydration dynamics during exercise. While systemic water loss has long been recognized as a hallmark of dehydration, localized changes can be detected in the oral cavity, highlighting the complex interplay between the hydration status and various physiological systems. The sensitivity of the oral mucosa to changes in hydration levels suggests its potential as a readily accessible target for assessing overall hydration status. The oral mucosa plays a pivotal role as a dynamic and interactive interface between the surrounding external environment and the physiological milieu of the body, making it particularly responsive to changes in hydration status.

The exercise-associated loss of oral mucosal moisture observed in the current study likely reflects a combination of factors, including reduced saliva production and increased evaporation rates due to heightened respiratory and metabolic activity during physical exertion. The redistribution of body fluids to active muscles and vital organs may further contribute to localized dehydration within the oral cavity [31]. Furthermore, the correlation between the post-exercise sweat rate and changes in oral mucosal moisture content provides evidence of the systemic nature of dehydration and its impact on oral mucosal hydration. This suggests that the loss of oral mucosal moisture may reflect the loss of body fluids during exercise; thus, quantifying oral mucosal moisture represents a noninvasive means of measuring overall hydration levels during exercise.

This study presents the novel finding that oral mucosal moisture may serve as an effective marker of dehydration status in athletes with inadequate fluid intake. Based on the current results, we recommend a cutoff value of 1.9 or 2.0 as the difference in oral mucosal moisture content needed for detecting dehydration. However, further investigation is required to refine this cut-off value and assess its efficacy in different age groups, sexes, environments, and exercise intensities. By elucidating the complex relationship between systemic and oral mucosal hydration, this study highlights the potential of oral mucosal moisture assessment as a valuable adjunct to traditional hydration monitoring methods.

Physiological, functional, and organic changes in the oral cavity due to exercise-induced dehydration have been previously reported. For example, increases or decreases in swallowing frequency [32], changes in taste [33], and salivary osmolality associated with exercise-induced dehydration [34] have been reported. They highlighted the potential of salivary osmolality as a marker of exercise-induced dehydration, although the interpretation of these results is limited. However, thirst sensation did not provide any predictive value. These results suggest that changes caused by dehydration are more likely to occur in the oral cavity. In addition to oral mucosal moisture, the inclusion of salivary osmolality and other parameters in the assessment may have the potential to improve detection.

The changes in s-IgA concentration and secretion rate observed in the present study provide insights into the physiological responses that occur during exercise-induced dehydration. s-IgA plays a central role in mucosal immunity, acting as the primary line of defense against pathogens in the oral cavity and upper respiratory tract. Therefore, changes in s-IgA levels can provide crucial information regarding the body’s immune status and ability to cope with exercise-associated stressors. Prolonged intense exercise reduces the secretory capacity of s-IgA, such as marathon running.

This study revealed a decrease in the IgA secretion rate and an increase in the concentration following exercise load. It was inferred that such fluctuations were one of the phenomena in which dehydration that occurred in the body was tied to and manifested as dehydration in the oral cavity. Dehydration during exercise results in fluid loss from the body, leading to changes in salivary composition and flow rate. Thus, when the salivary flow rate is reduced, the concentration of salivary components increases; consequently, the effects of dehydration cannot be overcome. In other words, the immunological quantification of saliva and other oral samples indicates that the presence or absence of dehydration should be considered when interpreting the increase or decrease in the concentration of biological substances, as biological substances may be concentrated with dehydration. In the present study, the s-IgA secretion rate was calculated to compensate for this effect. A reduced s-IgA secretion rate may weaken mucosal immunity because the body reallocates resources to prioritize thermoregulation and cardiovascular function over immune defense [35]. The alterations in s-IgA concentration and secretion rate observed in the present study provide insights into the intricate interplay between exercise-induced dehydration and immune response.

The present study is limited by the potential bias in participant characteristics and its observational field survey design, which lacks standardized conditions and a control group, thereby precluding a comparative study. The sensor used in this study was designed for use in a clinical setting, specifically in an indoor environment. To facilitate the widespread application of dehydration assessment in the context of outdoor exercise, it is essential to consider a multitude of variables, including human factors such as sex, age, fitness level, breathing pattern, circadian rhythm, training attire, dietary intake, and water intake; environmental factors, such as WBGT, wind direction and intensity, season, and weather; and exercise factors, such as exercise intensity and duration. It is essential to examine the impact of a multitude of variables, as they may vary under different conditions and environments. It is also important to consider the demographic distribution of participants. The subjects recruited for this study were not a demographically diverse sample (not a large number of children or adolescents), and they were a highly active population capable of running daily. This limits the generalizability of the findings as it is challenging to extrapolate from a sample of highly active individuals to a broader population, necessitating studies with larger, more diverse participant pools. The results of these measurements indicate that the device exhibited some technical sensitivity to the pressure and angle of contact with the surface in question. In other words, the control of the pressure inherent in capacitor sensors is a crucial factor influencing the outcomes, and the standardization of usage is also a significant consideration. Additionally, a detailed validation of the reliability and accuracy of capacitance sensor-based oral epithelial moisture meters is lacking. Further analysis is needed to establish the causality or correlation between changes in oral mucosal moisture content and exercise-induced dehydration, with caution against the overgeneralization of the results.

## 5. Conclusions

The results of the present study indicate the potential of oral mucosal moisture assessment as a noninvasive method for assessing exercise-induced dehydration status.

Further research is required to elucidate the causal relationship between moisture content in the oral mucosa and dehydration.

## Figures and Tables

**Table 1:** Exercise performance during 120 min long slow distance training.

Exercise Index	Value (Mean ± Standard Deviation)
Maximum heart rate (bpm)	178.2 ± 15.2
Running distance (km)	18.6 ± 2.0
Perceived exertion (Borg scale)	4.5 ± 1.3

**Table 2:** Dehydration assessment data before, during, and after 120 min long slow distance training.

Parameter	Pre-Exercise	Post-Exercise	Difference	During Exercise
Body weight (kg)	60.5 ± 12.1	59.4 ± 12.1	1.1 ± 0.5 *	
Fluid intake (L)				0.5 ± 0.2
Sweat rate (L/h)				0.77 ± 0.26
Saliva flow rate (mL/min)	1.0 ± 0.5	0.7 ± 0.5	0.3 ± 0.4 *	
Oral mucosal moisture	30.8 ± 1.3	29.5 ± 1.6	1.4 ± 1.6 *	

* *p* < 0.05; Comparison before and after LSD training using Student’s paired *t*-test/Wilcoxon signed-rank sum test.

**Table 3:** Secretory immunoglobulin A (S-IgA) absolute concentration and secretion rate before and after 120 min long slow distance training.

S-IgA	Pre-Exercise	Post-Exercise
Concentration (µg/mL)	371.7 ± 258.3	390.0 ± 196.4
Secretion rate (µg/min)	333.0 ± 240.4	264.6 ± 179.8 *

* *p* < 0.05.

**Table 4:** The results of a multiple regression analysis for sweat rate.

Outcome	*p*-Value
differences in oral mucosal moisture levels	0.02 *
body weight	0.05
gender	0.11
pre-training oral mucosal moisture content	0.33
differences in oral mucosal moisture levels × pre-training oral mucosal moisture content	0.56
differences in oral mucosal moisture levels × gender	0.69
differences in oral mucosal moisture levels × body weight	0.84

* *p* < 0.05.

## Data Availability

The data presented in this study are available upon request from the corresponding author, owing to privacy concerns.
